# A Rapid Method for the Determination of Pneumococcus Types

**Published:** 1918

**Authors:** 


### MEDICINE

---

*A Rapid Method for the Determination of Pneumococcus Types.* By O. W. H. Mitchel and Walden E. Muns, College of Medecine, Syracuse. Abs. from the *Journal of Medical Research,* Nov., 1917.

The authors offer a more rapid method of distinguishing pneumococcus types because methods now in use are too slow to make early recognition possible. Early differentiation is especially desirable since only the immune serum for Type I pneumococcus is of practical value.

The test devised consists in the detection of precipitinogen, derived from the pneumococci in the sputum. By this method there is no such delay as in the use of a mouse or in the securing of a culture. Type I may generally be recognized within an hour or two.

Results thus obtained in the laboratory have proved accurate. They have been checked by mouse inoculations.

*Technique.* — The sputum (5 cc.) is pipetted into a small mortar. Fine sand is added until a stiff mixture is obtained. The mixture is then ground for about three minutes until a gritty fluid paste results. 10 cc. of normal saline are added, two at a time. After thorough mixing, the sand is allowed to settle. After three or four minutes the dissolved sputum collects over the sand and is pipetted off into a centrifuge tube. A second fluid is obtained by mixing about 10 cc. of normal saline solution with the sand in the mortar and pipetting off into a centrifuge tube. This may be reserved for use in case of accident. Both solutions are centrifuged for from 5 to 10 minutes at 2,200 R.P.M.

Positive results have thus far been obtained from tests made with Types I, II, and III of anti-pneumococcic serum, furnished by the Division of Laboratories and Research, New-York State Department of Health. In testing for each type, 0.2 cc. of the serum is mixed with 1.0 cc. of the sputum solution. The tube should be carefully shaken and placed in a water bath held at 37° C. Many of the reactions appear almost at once, especially that of Type I. The mixture first appears slightly opalescent with a faint smoky " whirl " on shaking. (The " whirl " is due to finely divided particles of sputum.) The disappearance of the " whirl " seems to be the first indication of specific reaction. A very slight feathery precipitate may then be detected with the naked eye. With a lens this precipitate may sometimes be seen within a few minutes after the mixing. The feathery white flakes tend to drift to the bottom of the tube, where they collect in a typical white mass.

In a negative test there is no reaction of any kind and very little sedimentation. If sedimentation occurs, it appears as a nebulous grayish cloud at the bottom of the tube, remaining partly in suspension. Precipitate from specific reaction, upon shaking, breaks up into large particles, but sedimentation, when shaken, disappears through the fluid.

After two hours in the water bath, the tubes may be set away in the ice-box. Generally there is no reaction after three or four hours.

Sputa from 36 cases of well-developed pneumonia were examined. Although pneumococci of Type IV were isolated, no reaction could be obtained by any of 7 tests. Positive results were obtained in only 1 out of 4 tests for Type III. For Type I, however, positive results were obtained in 18 out of 19 tests. The 4 cases of Type II all gave positive results.

